# Buffalo Medical and Surgical Association

**Published:** 1884-08

**Authors:** Frederick Peterson

**Affiliations:** Secretary


### Society Reports.

### Buffalo Medical and Surgical Association.

*Adjourned Meeting, July 15th, 1884.*

Dr. F. W. Bartlett, President, in the Chair.

The minutes of the last meeting were read and approved.

Dr. Roswell Park was proposed and elected a member of the association.

Dr. Irving M. Snow applied for membership.

Dr. John H. Pryor read a very interesting paper on "The Status of the Midwife in Buffalo," in which he took the ground that she is utterly incapacitated for the practice of obstetrics and should be abolished altogether.

*Discussion*—Dr. Rochester had seen a great many atrocities committed by midwives. They keep patients in the same unclean clothes for a week and do not know the use of water. They go from one patient to another, carrying diseases of various kinds. This condition of things is much worse here than in Europe, where a certain amount of education is required. Some women cannot be convinced that a midwife is not better than a man, and she is much cheaper. There should be some provision for the attendance of the poor by physicians at a moderate compensation; for instance, by dispensary service.

Dr. Coakley would add to some of the abuses practiced by midwives that of the use of alcohol in almost every case, without regard to the patient. He remembered a woman in another city who had the largest obstetrical practice in the city. She received $10 per case. She had attained to considerable dexterity in the treatment in some of the concomitant affections, such as the prevention of mammary abscess by manipulation and inunction.

Dr. Hartwig thought it as much a political as a medical question, the disposition of the midwife. If a patient desires aid, let her seek it where she will. This is a free country. In Germany they are also, at this time, discussing this matter, although there they are educated to a very high degree. They are especially taught the diagnosis of positions, and to know when to call in the doctor. The foreigners in America know very well the difference between the educated midwife of Germany and the self-made one of America. The real danger is in their freedom, their being responsible to no one. He mentioned one midwife who has had 1,000 successive successful cases, among which were fifty footling cases, one placenta prævia, fifty placentas to remove by hand, and fifty cases of cross-presentation, where she had to turn. Only three times did she call a doctor to apply forceps. No mother nor child died during confinement. This he considers true. He thinks there is a place for the midwife in this country. She washes the child, makes soup, etc., which doctors cannot do. Parturition is a physiological process, and the midwife is the one to oversee it.

Dr. Weil stated that physicians already charge sufficiently small prices, the old ones, especially, so their services are within the reach of the poor. He mentioned instances.

Dr. Ring thought every woman entitled to treatment from a physician, because she is a woman and a mother. We owe it to her to abolish the midwife. Fees should be lowered to suit the conditions of patients.

Dr. Stockton formerly held the same bad opinion of midwives as the essayist, but he has changed his mind. He also knows of a midwife who has had 1,000 cases without a death, and has heard other physicians speak of similar cases. He thinks obstetrics should be the work of the midwife.

Dr. Hopkins was amazed to learn that the doctors take charge of but one-third of the births of this city. He could scarcely believe that the average midwife attended a thousand cases without death of a mother. He believed no physician present had had such experience. He thought the question of disposing of the midwife one of great importance, and that the society should could continue to study the matter, as one evening's discussion would not be sufficient. We might address a memorial to the State Society relative to this subject, and learn if in other parts of New York the midwives had two-thirds of the obstetrical cases.

Dr. Van Peyma has had much to do with midwives in his practice at East Buffalo. Although the paper showed the necessity for reform, he thought it one-sided. He himself had great respect for the midwives with foreign diplomas. In his own family he would certainly prefer a midwife to a district physician. He thinks the statistics given the doctors by midwives not at all reliable. They call in a doctor in their bad cases, and he signs the death certificates; hence, no deaths occur in their practice. As to the case referred to by Dr. Pryor, where a midwife was washing out the vaginas of nine patients (one having puerperal fever) with the same syringe, he considered her one of the best midwives at East Buffalo.

Dr. Cronyn considered the midwife very useful. He has been brought much into contact with them. Like the essayist he had also noticed that they were all widows. They probably had followed this practice because of their own experience. He knew of one woman who had attended 5,000 cases of labor in twenty-five years. She is very ignorant. The following dialogue once took place between them: "Where did you learn the trade?" "I have read six books." "Who wrote them?" "My husband." Midwives should be admitted to lectures in this branch of medicine at our colleges. He has found many of them cleanly and many far from that. Some are acquainted with antiseptics.

Dr. Mann thought it would be much better to do away with the midwife and substitute the physician, but it was not practicable. Since it seems we must have the midwife, we must endeavor to make her good. It is the duty of the profession to educate her.

Dr. Bartlett mentioned several cases of maltreatment of patients by midwives and thought Dr. Hartwig's statistics wholly untrustworthy. He has heard of a number of physicians who charge less than the ordinary fee for obstetrical attendance in order to increase their practice. Some women prefer midwives, he thinks, because they are afraid the doctors will employ instruments in their hurry to get through. He thinks the midwife uses too much (and too ignorantly) ergot.

Dr. Pryor, in closing the discussion, said he had, in his paper, spoken of the one hundred midwives of this city collectively. He described the average midwife. The various speakers who had extolled this creature did so merely from their one-sided experience with the single ones with whom they had been brought in contact. He would not be satisfied unless the society acted upon the suggestions of his essay. If Dr. Van Peyma considered the example he mentioned one of the best midwives at East Buffalo, we had a good criterion from which to form an opinion of the rest.

On a motion being carried to appoint a committee to consider this matter and report subsequently, the Chair named Drs. Hopkins, Ring, Hartwig, Pryor and Van Peyma to serve as such.

*Prevailing Diseases*—Dr. Hartwig reported measles and dysentery. Dr. Stockton reported whooping-cough. Dr. Van Peyma reported measles. Dr. Cronyn reported scarlet fever. Dr. Bartlett had noticed a marked increase in whooping-cough.

*Reports of Committees*—Dr. Coakley, chairman of the committee appointed to determine upon the plan of dividing the society into sections, stated that no formal meeting had been held, but that it had been agreed by those who favored this division to meet at the office of the President at an early day for organization.

*Miscellaneous Business*—A resolution was proposed to the effect that the BUFFALO MEDICAL AND SURGICAL JOURNAL be requested to publish the proceedings of this association regularly, and that the Secretary be required to transmit to the editors his record of each meeting for their columns.

Dr. Van Peyma, one of the Editors, remarked that he had failed to notice that the proceedings had not been regularly printed in the JOURNAL and that hereafter they should appear.

Dr. Hopkins thought the thanks of the editors due the society for calling their attention to the fact that the Secretary's report had not appeared in their columns for a long time.

The resolution was passed.

A letter of resignation from Dr. W. S. Tremaine was read.

A motion to lay the letter upon the table was carried, and the secretary was instructed to write the gentleman a note, calling his attention to Art. ii, Sec. 2, of the Constitution and By-Laws.

The meeting then adjourned.

FREDERICK PETERSON, M. D., *Secretary.*

